# Learning from the COVID-19 pandemic for future epidemics and pandemics preparedness and response in Guinea: Findings from a scoping review

**DOI:** 10.3389/fpubh.2022.879850

**Published:** 2022-10-17

**Authors:** Delphin Kolié, Fatoumata Namaren Keita, Alexandre Delamou, Jean-Paul Dossou, Wim Van Damme, Irene Akua Agyepong

**Affiliations:** ^1^African Centre of Excellence in the Prevention and Control of Communicable Diseases, University of Conakry, Conakry, Guinea; ^2^Ministry of Health, Centre National de Formation et de Recherche en Santé Rurale de Maferinyah, Forécariah, Guinea; ^3^Centre de Recherche en Reproduction Humaine et en Démographie, Cotonou, Benin; ^4^Public Health Department, Institute of Tropical Medicine, Antwerp, Belgium; ^5^Ghana College of Physicians and Surgeons, Faculty of Public Health, Accra, Ghana; ^6^Research and Development Division, Ghana Health Service, Dodowa Health Research Centre, Accra, Ghana

**Keywords:** Guinea, policy decisions, health system preparedness and response, COVID-19, resilience

## Abstract

The outbreak of the novel coronavirus (SARS-CoV-2) in December 2019 prompted a response from health systems of countries across the globe. The first case of COVID-19 in Guinea was notified on 12 March 2020; however, from January 2020 preparations at policy and implementation preparedness levels had already begun. This study aimed to assess the response triggered in Guinea between 27^th^ January 2020 and 1^st^ November 2021 and lessons for future pandemic preparedness and response. We conducted a scoping review using three main data sources: policy documents, research papers and media content. For each of these data sources, a specific search strategy was applied, respectively national websites, PubMed and the Factiva media database. A content analysis was conducted to assess the information found. We found that between January 2020 and November 2021, the response to the COVID-19 pandemic can be divided into five phases: (1) anticipation of the response, (2) a sudden boost of political actions with the implementation of strict restrictive measures, (3) alleviation of restrictive measures, (4) multiple epidemics period and (5) the COVID-19 variants phase, including the strengthening of vaccination activities. This study provides several learning points for countries with similar contexts including: (1) the necessity of setting up, in the pre-epidemic period, an epidemic governance framework that is articulated with the country's health system and epidemiological contexts; (2) the importance of mobilizing, during pre-epidemic period, emergency funds for a rapid health system response whenever epidemics hit; (3) each epidemic is a new experience as previous exposure to similar ones does not necessarily guarantee population and health system resilience; (4) epidemics generate social distress because of the restrictive measures they require for their control, but their excessive securitization is counterproductive. Finally, from a political point of view, decision-making for epidemic control is not always disinterested; it is sometimes rooted in political computations, and health system actors should learn to cope with it while, at the same time, safeguarding trusted and efficient health system responses. We conclude that health system actors anticipated the response to the COVID-19 pandemic and (re-) adapted response strategies as the pandemic evolved in the country. There is a need to rethink epidemics governance and funding mechanisms in Guinea to improve the health system response to epidemics.

## Introduction

An unprecedented outbreak of a novel coronavirus (SARS-CoV-2) that emerged in China in December 2019 and rapidly spread to 114 countries across the globe by 11 March 2020 resulted in the World Health Organization's (WHO's) declaration of a pandemic (1). In Africa, the spread of SARS-CoV-2 was initially centered in the northern (Algeria, Egypt, Morocco) and southern (South Africa) regions, where significant increases in the number of cases and deaths were reported from March 2020 onwards (2). In late April 2020, however, the virus had almost spread throughout the continent, and most African countries were experiencing community transmission (2). Meanwhile, the capacity of the region's health systems to effectively implement pandemic containment measures—travel restrictions, increased testing, contact tracing, isolation of cases and quarantine of contacts—was apparently sparse (3–5). Moreover, the ability of African economic systems to resist and recover from these containment measures represented another tricky situation (3, 4). These challenges led to the international prediction of a drastic effect of the pandemic in Africa (6).

In Guinea, the first case of the novel coronavirus disease (COVID-19), imported from Europe, was reported on 12 March 2020 and the first death on 14 April 2020. At the time the pandemic hit Guinea, the country was undergoing a constitutional reform, with a background of socio-political crises. Moreover, no formal national framework for epidemic-preparedness and response existed at the pandemic onset, although the National Agency for Health Security (ANSS)—the former national Ebola response committee—had gained more power and influence in epidemic management in the past years, since its establishment in June 2016 (7). Therefore, the pandemic declaration resulted in a power struggle among several actors of the health system in Guinea (8, 9). These contextual factors certainly contributed to hindering the gains of the post-Ebola health system strengthening reforms for the initial COVID-19 preparedness and response (10, 11). From mid-April 2020 onwards, for example, a steep increase in cases was observed in Guinea, compared with Sierra Leone and Liberia, which had also been confronted with the 2014/2016 Ebola epidemic (8, 12). Nevertheless, in the same period, the number of reported deaths was much lower in Guinea compared with Sierra Leone, Liberia and many other west African countries (12).

Some authors have documented Guinea's response to the COVID-19 pandemic (8, 9, 12–15). However, these studies focused on the country's early response while the pandemic was unfolding. At the time of writing this paper (November 2021), Guinea has experienced three epidemiological waves of the COVID-19 pandemic; the first wave being the longest (from March 2020 to January 2021) and the last the deadliest (43 deaths per month on average compared to 7 and 18 respectively during the first and second wave). It is, therefore, necessary to establish a broader picture of the country's experience during the first and subsequent waves of the pandemic.

The national healthcare system is essentially based on the public service with three distinct levels of health services provision: primary, secondary and tertiary. The primary level includes 410 health centers; the secondary level comprises 33 districts hospitals, and 7 regional; and the tertiary level is composed of 3 national hospitals. Households constitute the primary source of health financing through direct payment, with 62% of expenditure, followed by the external funding from multilateral cooperation (27%). The share of the Ministry of Health's budget in the national budget remained below 3% between 2010 and 2014, before rising to 8% in 2016, after the Ebola epidemic.

In terms of clinical management of epidemic and pandemic prone diseases, the country has 33 epidemic management centers, distributed among the health districts. At national level, several institutions have been capacitated in the post-Ebola context to improve epidemics preparedness and response in the country. These institutions include the national directorate for epidemiology and disease control (DNELM), the national institute of Public Health (INSP), and the national health security agency (ANSS), with overlapping functions in the epidemics surveillance and control. However, from 2017 onwards, the ANSS has gained more Leadership in epidemics surveillance and control, including sporadic cases of human anthrax, measles, varicella, and yellow fever (7). The ANSS has deconcentrated structured integrated to regional (regional alert and response team) and district (district team of alert and response) health systems. The ANSS is technically and financially supported by the Ministry of Health and development partners including the world health organization (WHO), the center for disease control and prevention of Atlanta (CDC), and the World Bank (7).

Therefore, this study aimed to describe policy decisions to the preparation and response to the COVID-19 pandemic from January 2020 to November 2021, community responses to these decisions. Such findings would provide lessons for management of future epidemics and pandemics in Guinea, and countries with similar context.

## Methods

### Study design

This study was conducted using the scoping review guide developed by the Joanna Briggs Institute (JBI) (16). We opted for this study design because of the scope of our research questions.

This scoping review was conducted using the five stages for scoping review outlined by the JBI manual: (1) identification of research question, (2) identification of relevant studies, (3) study selection, (4) data charting, and (5) summarizing and reporting results.

### Identification of research questions

For this review, two main research questions were identified:

1. What policy response was undertaken before, during and after each of the COVID-19 waves registered in Guinea over the period Jan 2020 to Nov 2021?
2. How has the national community reacted to the policy decisions?

### Search strategy

A documentary search plan was developed to integrate five different sources: websites of national institutions, PubMed search engine, Google search engine, Media content search and consultation with key informants for gray literature sources. For each of these sources, a specific search strategy was applied to facilitate the generation of relevant information.

- The website of the national agency for health security (ANSS) was browsed for the period between January 2020 and November 2021 to identify policy documents (decrees, acts, etc.), and relevant information on the pandemic response (debriefing, meeting notes, etc.).
- The second search technique consisted of contacting key informants to help get policy documents that were not found on the ANSS website. A Snowball and citation tracking techniques were used for identifying and asking for additional policy documents. Key informants contacted included officials of the ANSS, international migration organization, world health organization, national public health institute, and the scientific committee for COVID-19 response.
- Three search terms were used for the PubMed search: (1) Coronavirus OR COVID-19 OR Novel Coronavirus; AND (2) preparedness OR response OR governance OR management; AND (3) Guinea. For the Google search, several search strategies, containing multiple combinations of the above search terms, were created. In order to ensure consistency across searches and effective time management, only the first five pages of each search were screened (17).
- For the Media content search, the Factiva media database (https://www.dowjones.com/professional/factiva/) was used. This search strategy required defining in advance: (1) search terms, (2) period of the search, (3) targeted media and (4) search Language (French and English). We replicated the search terms used for articles identification on PubMed. This search covered the period between January 2020 and November 2021. Finally, media targeted for our search included Africaguinee.com, Guinée Matin, Guinee7.com, Journal de Conakry.com, Jeune Afrique, Agence de Presse Senegalaise, Agence Ivoirienne de Presse, L'intelligent d'Abidjan, Agence France Presse, BBC, RFI.fr, and FoxNews.

### Studies selection

Our search results were cleaned for duplicates and two members of our research team (DK and FNK) independently screened studies using the eligibility criteria described in Table 1. The snowball and citation tracking techniques were used to identify additional articles or documents to be added to selected studies. Discrepancies in study selection processes were resolved by a discussion between the two reviewers with the assistance of another team member (AD).

**Table 1:** Eligibility criteria for selection of studies.

**Inclusion criteria**	**Exclusion criteria**
Full text written in French or English	Unavailable in French or English
Most current version of the document	Document was a draft or summary version or has been replaced with another document
Articles, reports, opinion papers, workshop summaries, briefings, commentaries, blogs, newspaper articles	Abstracts
Include interventions or strategies for the COVID-19 response in Guinea	Include interventions or strategies for the COVID-19 response for other contexts different from Guinea

**Figure 1:**
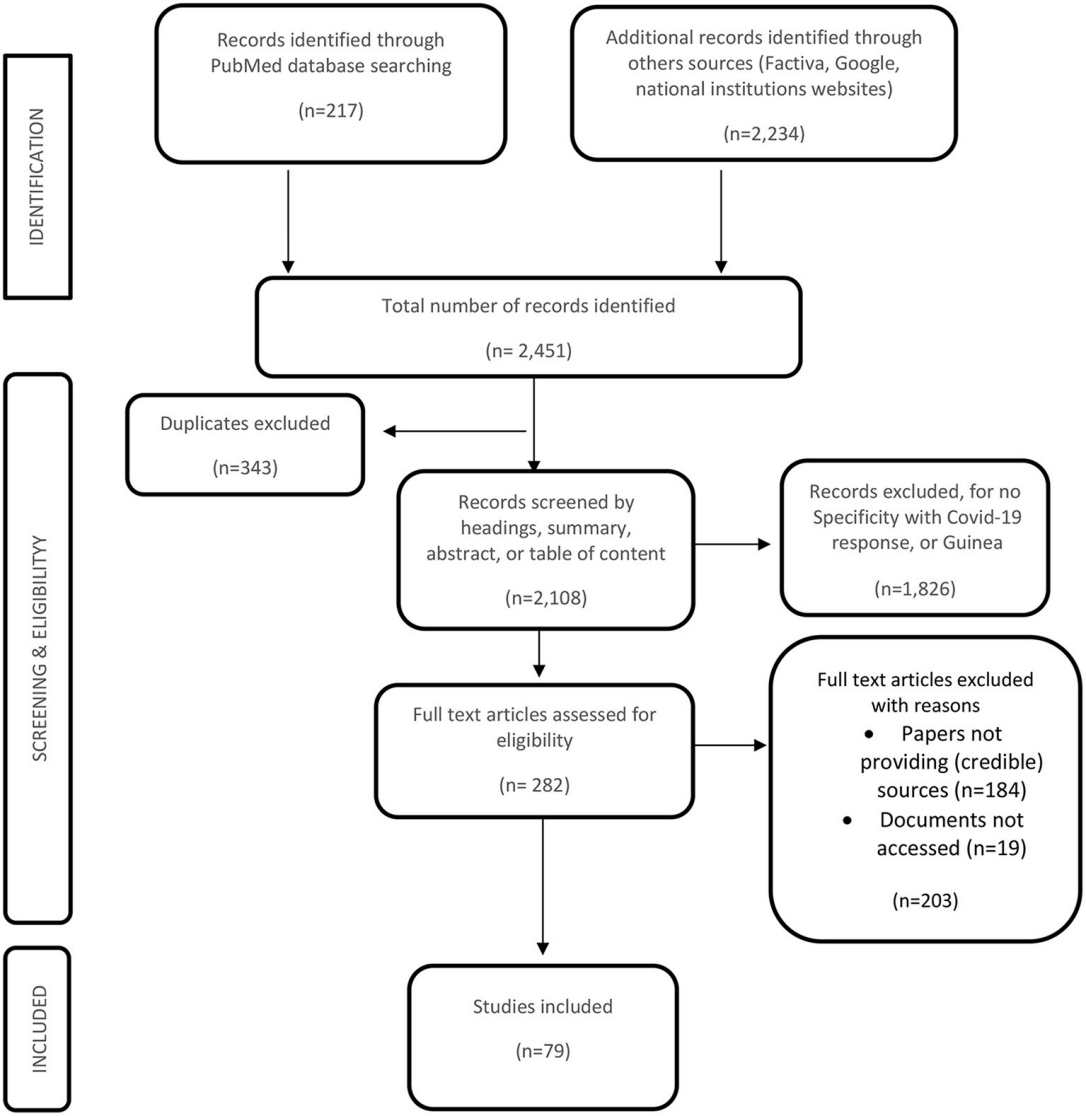
Study inclusion flow chart.

In total, 79 studies were included in this review. Figure 1 shows the search description flow chart of included studies. Table 2 shows the detailed characteristics of studies included and also sources used for their selection. Policy documents represented 46.25% of data sources included in the study followed by media content (38.75%).

**Table 2:** Documents characteristics.

**No**.	**Document**	**Date of publication**	**Search strategies used to identify document**
	**Policy documents**		
1	Technical guidelines for managing the risk of spreading the new coronavirus	27 January 2020	Consultation with policy actors
2	Coronavirus threat management Guidelines	12 February 2020	Consultation with policy actors
3	National COVID-19 Preparedness and Response Plan	19 February 2020	Consultation with policy actors
4	Setting-up of six technical commissions for the response to COVID-19	14 March 2020	Consultation with policy actors
5	Presidential decree on the measures to strengthen Coronavirus management	21 March 2020	Targeted websites, consultation with policy actors
6	Declaration of a state of health emergency	26 March 2020	Targeted websites, consultation with policy actors
7	Act of reinforcement of the prerogatives of the national agency for health security on the management of the COVID-19	27 March 2020	Consultation with policy actors
8	Address to the Nation by the President of the Republic on new measures against COVID-19 in Guinea	30 March 2020	Targeted websites
9	Communiqué on the mobility of people from Conakry to the countryside	31 March 2020	Targeted websites
10	COVID-19 management technical guide	April 2020	Consultation with policy actors
11	Plan of economic response to the health crisis of COVID-19	2 April 2020	Google search, consultation with policy actors
12	Creation of the scientific council (task force) of response to COVID-19	10 April 2020	Consultation with policy actors
13	Decree on the reinforcement of the state of emergency measures	13 April 2020	Consultation with policy actors
14	Stop COVID-19 in 60 days Community Response Strategic Plan	May 2020	Consultation with policy actors
15	Plan to strengthen the health system and resilience for the continuity of services in the COVID-19 context	19 May 2020	Google search, consultation with policy actors
16	Address to the Nation of the Head of State (Alpha Condé)	15 July 2020	Targeted websites
17	Circular note on the end of home confinement	27 July 2020	Targeted websites, consultation with policy actors
18	Note on the celebration of the Tabaski festival on July 31 2020	23 July 2020	Targeted websites
19	Note on the implementation of COVID-19 tests for air travelers	27 July 2020	Consultation with policy actors
20	Directive on body transfer authorization	19 August 2020	Targeted websites
21	Circular note on the identification and follow-up of COVID-19 contacts	20 August 2020	Targeted websites
22	Guidelines of the reopening of training institutions (schools)	October 2020	Consultation with policy actors
23	Guidelines for Reopening Places of Worship	October 2020	Consultation with policy actors
24	Active case-finding strategy for COVID-19 coupled with sensitization in Guinea: Stop the COVID-19 “Let's get screened”	October 2020	Consultation with policy actors
25	Guideline for the systematic performance of antigenic rapid diagnostic tests in Guinea	8 October 2020	Targeted websites, Consultation with policy actors
26	Guideline for the systematic realization of antigenic rapid diagnostic tests in Guinea	September 2020	Consultation with policy actors
27	Government press release on guidelines for the application of restrictive measures	18 September 2020	Targeted websites
28	Alleviation of restrictive measures in the transport, tourism, sports and culture sectors	25 September 2020	Targeted websites
29	National vaccination plan against COVID-19 in Guinea	December 2020	Consultation with policy actors
30	Active Case Finding and Outreach Strategy	1 December 2020	Consultation with policy actors
31	Decentralization of COVID-19 response through the strengthening of local coordination and intensification of community contacts follow-up	1 December 2020	Consultation with policy actors
32	Setting-up of the steering committee for the introduction of COVID-19 vaccines	2 December 2020	Consultation with policy actors
33	Temporary authorization for the use of Sputnik V vaccine in Guinea	21 January 2021	Targeted websites
34	Government guidelines on the management of the Ebola virus disease in Guinea	16 February 2021	Targeted websites
35	Decision on the deployment of national and international experts to N'zérékoré for the joint response to Ebola and COVID-19	19 February 2021	Targeted websites
36	Plan to accelerate vaccination against COVID-19 in Guinea, “stop COVID-19, let's vaccinate”	September 2021	Consultation with policy actors
37	Introduction of the “Vaccination pass”	1 September 2021	Targeted websites
	**Research articles**		
38	First-line response to COVID-19: community health centers and doctors' offices in Guinea (9)	April 2020	Google search
39	COVID-19 in Guinea: The first line of health care in South and North get ready for action! (15)	May 2020	Google search
40	Willingness to comply with physical distancing measures against COVID-19 in four African countries (13)	September 2020	PubMed search
41	Tackling the COVID-19 pandemic in West Africa: Have we learned from Ebola in Guinea? (8)	December 2020	PubMed search
42	Evolution of the COVID-19 pandemic over 6 weeks in four French-speaking countries in West Africa (12)	January 2021	PubMed search
43	Ebola and COVID-19 in DR Congo and Guinea (18)	April 2021	PubMed search
44	Ebola Outbreak amid COVID-19 in the Republic of Guinea: Priorities for Achieving Control (19)	April 2021	PubMed search
45	The COVID-19 pandemic in francophone West Africa: from the first cases to responses in seven countries (14)	August 2021	PubMed search
46	Marburg virus amidst COVID-19 pandemic in Guinea: Fighting within the looming cases (20)	September 2021	PubMed search
47	Guinea's response to syndemic hotspots (7)	October 2021	PubMed search
48	Will Guinea's coup interrupt the country's health responses?	September 2021	Google search
	**Media content**		
49	Elections held in Guinea despite COVID-19	19 March 2020	Factiva software search
50	In Guinea, Alpha Conde plays his cards right	21 March 2020	Factiva software search
51	Alpha Conde makes a gesture to the Army on the eve of the elections	March 2020	Factiva software search
52	Ali Baba Foundation donate COVID-19 test in Guinea	April 2020	Factiva software search
53	Guinea: Deadly protests in several cities against police blockades	May 2020	Factiva software search
54	COVID-19: un médecin guéri de la pandémie témoigne	30 May 2020	
55	Start of the 2020 university year: our findings in Sonfonia and Mahatma Gandhi of Lambanyi	October 2020	Factiva software search
56	The United Arab Emirates donates field hospital to Guinea	November 2020	Factiva software search
57	Draft budget 2021: the government announces a budget of more than GNF 27 600 billion	October 2020	Factiva software search
58	Guinea: Opposition protesters injured (witnesses, hospital source)	October 2020	Factiva software search
59	Back to school: DPE N'Zérékoré gives instructions to school administrators	October 2020	Factiva software search
60	National Assembly: deputies adopt the LFR 2020 which amounts to about GNF 27 000 billion	November 2020	Factiva software search
61	Jacques Gbonimy, president of the UPG: ‘COVID-19 has become a political disease in our country'	November 2020	Factiva software search
62	Guinea bans rallies citing virus, opposition cries foul	November 2020	Factiva software search
63	Fight against COVID-19: three doctors of the RUSAL company rewarded by the structure Katala 224	November 2020	Factiva software search
64	Council of Ministers: Here are the full minutes	November 2020	Factiva software search
65	COVID-19: President Alpha Conde extends the State of Emergency for 30 days	November 2020	Factiva software search
66	European Union and Federal Republic of Germany Provide COVID-19 Protection Kits to Guinean Government	November 2020	Factiva software search
67	Council of Ministers: here are the full minutes (Press release)	November 2020	Factiva software search
68	Fight against COVID-19: Health providers announce a strike from November 13	October 2020	Factiva software search
69	The children must return to school, but the recipes of the past are no longer	October 2020	Factiva software search
70	Impact of COVID-19 in Guinea: more than 17 billion in losses suffered by the owners of places of leisure	October 2020	Factiva software search
71	Receipt in Conakry of 11 360 doses of Ebola vaccine by February 22, 2021	February 2021	Factiva software search
72	Launch of the vaccination campaign against COVID-19	March 2021	Factiva software search
73	Reception this Saturday, 6 March 2021 at 22:03 of 200 000 doses of Sputnik V vaccine	March 2021	Factiva software search
74	Press release on the vaccination against COVID-19 in Conakry	March 2021	Factiva software search
75	Guinea's school and the COVID-19 pandemic	March 2021	Google search
76	Communiqué from the Ministry of Health on the use of the batch of 69 000 doses of AstraZeneca vaccine received in Guinea on 29 March 2021	March 2021	Factiva software search
77	As part of the COVAX initiative, the Guinean government received its first batch of the AstraZeneca vaccine on Sunday, April 11, 2021. Composed of 194 400 doses	April 2021	Factiva software search
78	Receipt of 300 000 doses of #Sinovac vaccine purchased by the Guinean government to increase the country's vaccine capacity and allow many people to be vaccinated	April 2021	Factiva software search
79	Communiqué on the administration of the second dose of Sputnik V vaccine	April 2021	Factiva software search

### Data analysis

The audio recordings from the national press were completely transcribed. Content analysis of these transcripts, media reports and policy documents were done manually according to a code grid. We compared the data from the different sources (media content, audio records, policy documents) to triangulate the data and thus strengthen the internal validity (credibility) of the findings. We addressed inter-coding bias by having two researchers coding the material and allowing for adjustment upon a team consultation in association with a third researcher.

## Results

**Figure 2:**
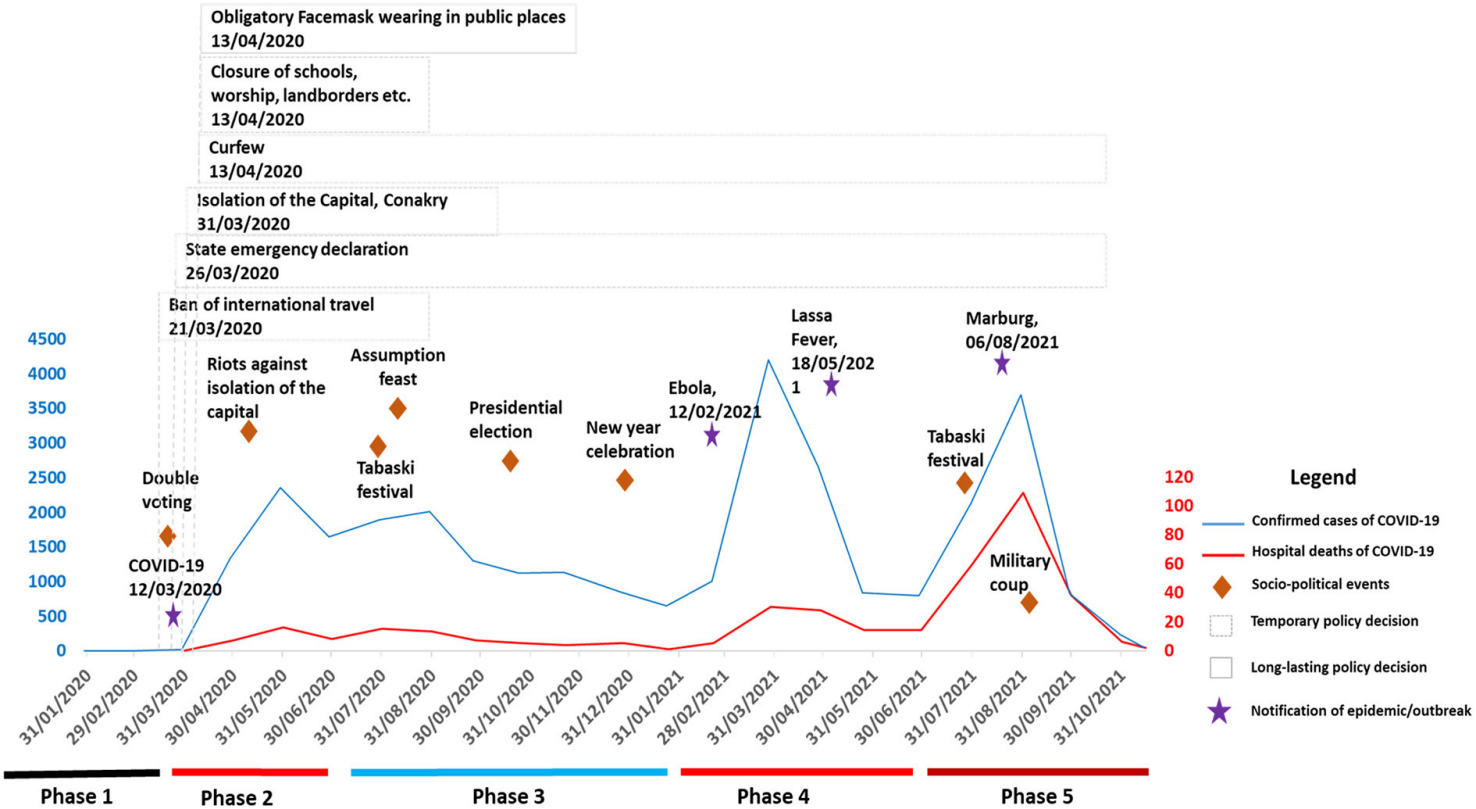
Chronology of key events during the preparation and response to the COVID-19 pandemic in Guinea, January 2020 to November 2021.

The chronology of events pertaining to the COVID-19 response can be divided into five major phases or periods (described below) according to the national dynamics of the response: policy action or inaction, extensive nature of decisions or not, and the epidemiological context (increased in cases notification or not) (Figure 2).

### Phase 1: The anticipation of the pandemic response, 27 January to 20 March 2020

On 9 January 2020, the WHO declared the emergence of a novel coronavirus (SARS-CoV-2) in China. A few days after this event (20 January 2020), the ANSS started communication on the disease. Posts on the ANSS website indicated that this early communication aimed to assure the population that the health authorities were closely following the chronology of events related to this new disease and that, precautions were being taken to anticipate the health system response. On 28 January 2020, upon the demand of the ANSS, several national (Ministries of Health, Livestock, Transport, Environment and Water and Forestry) and international (WHO, The International Organization for Migration (IOM), the Centers for Disease Control and Prevention (CDC), Médecins Sans Frontières (MSF), Expertise France, etc.) actors met to evaluate the risk of the disease spreading in Guinea. During this meeting, an *ad hoc* committee was set up and entitled, among others, to vulgarize the symptoms and mode of transmission of the disease, and elaborate guidelines for identifying and managing suspected cases. The WHO statement of 30 January 2020 classifying the virus as a threat to human health reinforced the health actors in their approach of anticipating the country's response. Explicably, the WHO declaration triggered the development of the COVID-19 preparedness and response plan (February 2020) [Ministère de la Santé: (21)]. This plan, similar to the Ebola response strategy, was built around seven strategic axes— (1) epidemiological surveillance, (2) laboratory, (3) clinical care, (4) information and communication, (5) logistics, (6) coordination and (7) technical assistance—each of which incorporated two key phases of the pandemic control: alert and response. Measures undertaken in the alert phase, such as strengthening preventive measures at the 44 potential entry points primarily identified (airports, seaports, mining ports, land borders, etc.) intended to avert the introduction of the virus in the country [Ministère de la Santé: (21)]. Meanwhile, the response phase sought to limit the spreading of the virus and included measures such as the reinforcement of diagnostic capacities, training of healthcare providers, dignified and secure burial, and improvements of clinical care conditions through the rehabilitation of epidemic treatment centers (21).

However, some challenges that arose in this period should be noted. First, the cost of implementing this plan, estimated at USD 47 million (Currency of the United States of America), was not secured. At the same moment, the government increased military salaries by 20% (16 March 2020) (22). This certainly impacted the earlier national response to the pandemic. For instance, a situational analysis undertaken in March 2020 showed that the health system was unprepared to deal with this new crisis. In fact, the analysis showed important insufficiencies in terms of the availability of supplies (masks, gloves, medicines, etc.) and intensive care equipment, the inadequacy of epidemic treatment centers and poor awareness of healthcare workers. Moreover, the COVID-19 threat Management Guidelines, released in February 2020, indicated that in February 2020 itself, more than 15 000 entries were recorded at the national airport. Meanwhile, only three laboratories were available to test for COVID-19; making it challenging to test all suspected cases at arrival or ensure a 14-day follow-up, as planned in the COVID-19 preparedness and response plan of February 2020 (23). Nevertheless, these national laboratories had the necessary equipment for the diagnosis of pathogens such as SARS-CoV-2 and Ebola (24). Also, the country had nearly 2,000 health workers trained in community-based epidemiological surveillance (21).

The news of the devastating effects of the disease in Europe and Asia created fear and panic among part of the population, prompting their reactions to call for implementing social restrictive measures and postponing campaigns and elections (scheduled between 15 February and 23 March 2020). A health official interviewed on local media said ‘we can't hold elections while there is a pandemic. We have officially recorded two cases; it would be taking too much risk to go to these elections (25).

On 21 March 2020, a presidential decree was broadcasted, banning international travel, mass gathering (more than 20 people) and recommending people freshly coming from Europe and Asia to self-quarantine for 14 days. At the same time, however, legislative and referendum elections were maintained for 22 March 2020 as well as political campaigns (26).

### Phase 2: Sudden political action and implementation of strict restrictive measures, 21 March to 14 July 2020

By 31 March 2020, the country accounted for a total of 16 cases and zero death of COVID-19. Thereafter, we observed an explosion of cases with 1,335 new infections and 7 deaths recorded on 30 April 2020. By the end of May 2020, the country reached the first peak of 2355 new infections and 16 deaths. In response to this, the government established three sets of decisions: (1) social restrictive measures, (2) the pandemic's governing framework and (3) initiatives intended to mitigate the pandemic's effects.

#### Social restrictive measures: The one-way response strategy

At this stage of the pandemic response, several restrictive measures—similar to most western countries confronted with the acute phase of the disease—were established, giving the impression that the pandemic response strategy is unique regardless of the context. This period was also dominated by the rise of societal responses to the pandemic control decisions.

On 30 March 2020, President Alpha Conde, in a solemn speech, called on the Guinean people to take a patriotic surge against the rapid increase in cases and the spread of the epidemic beyond the capital and four rural health districts. Referring to this epidemiological context and the WHO prediction on Africa, he declared a state of emergency and announced a set of social restrictions measures until 15 May 2020: closure of schools, land borders, worship places, bars and other places of recreation; a reduction on the number of people allowed on public transport; and a curfew from 9 p.m. to 5 a.m. The government also declared the compulsory wearing of facemasks from 18 April 2020 onwards, and that offenders would be prevented from movement and would be charged a disobedience fee of USD 3. On 31 March 2020, the Ministry of Health (MoH) announced the isolation of the capital, Conakry, thus banning domestic transport toward the countryside.

Nevertheless, some of these decisions generated subsequent tensions between actors, including community resistance; and human and economic consequences. For instance, the limitation of the number of passengers in public transportation led to a 50% increase in fares (27). Moreover, following the setting up of military checkpoints to ban domestic travel toward the countryside, riots and protests broke out on 12 May 2020 in cities surrounding the capital (Dubreka and Coyah) leading to the death of five people (14, 28).

Although the contribution of the security department has been essential in the pandemic response— transporting medical supplies, mobilizing military health personnel and facilities for managing COVID-19 cases, and implementing policy decisions—some tensions were observed between the security department and the transporters' union. These tensions were linked to the setup of several “unnecessary” checkpoints and their implication on citizens' movements (29). Furthermore, several religious leaders and communities reacted to the closure of mosques arguing that this was a violation of their faith (9). Finally, an estimate of GNF 17 billion (GNF= Guinean currency) in economic losses was revealed by an official of “the Guinean Association of bar, motel, restaurant and nightclub owners” 6 months after the pandemic control measures were adopted in Guinea (30, 31). This loss was likely sustained by the prolonged closure of public places (31).

#### Organization of the health system response

This period consisted of establishing governing bodies and standards adapted to the challenges encountered in the response to the first wave. This included establishing the Leadership of the pandemic response and the (re-)organization of care delivery for interrupting the transmission chain.

##### The setting up of a governing framework: The repositioning struggle

Centralization of power and decision-making in the response to the first wave was originally considered (8, 9). On 27 March 2020, a presidential decree mandated the ANSS as the leading body of the pandemic response. Some national institutions considered it unacceptable to be overhung by the ANSS in their field of expertise (9). Others, such as teaching hospitals managers, threatened to withdraw from the fight against the pandemic and launched a strike after the ANSS recruitment of 350 medical students and their deployment in the pandemic treatment centers –instead of senior medical doctors and trainees in teaching hospitals (32). Furthermore, the MoH, the actual affiliated institution of the ANSS, showed its disagreement with this decision, arguing that it was not consulted beforehand. This situation prompted the President to initiate a series of dialogues and consultations with the various protagonists (about 30 people including the Minister of Health and the ANSS General Director) and to make them swear “to serve the Guinean people, and work together to fight the invisible enemy” (33).

In the days that followed, the pandemic response framework was established to involve a large range of national and international actors. This framework also included the scientific committee created by a presidential decree (10 April 2020) and composed of 17 national public health experts and academics with the mandate to monitor, analyse and advise the government on the pandemic response.

##### Care delivery organization for interrupting the transmission chain: Continuous adjustment of policy decisions

Strategies for interrupting the chain of community transmission were planned early in the pandemic preparedness and response strategy. However, their implementation experienced a slow pace. As COVID-19 cases increased in April 2020, several measures were taken to readjust the testing, contact tracing and isolation strategies.

First, the testing strategy of February 2020 identified three national laboratories in charge of performing RT-PCR antigen tests for COVID-19 suspected cases. Understandably, no rapid diagnostic antigen tests (RDTs) was available at the onset of the pandemic in Guinea, at least until the first donation of 20 000 COVID-19 antigen tests by a humanitarian Foundation on 1 April 2020 (34).

However, as community transmission intensified and as more RDTs became available from mid-April 2020 onwards, several changes in testing strategy were observed including decentralizing sample collection sites in Conakry (the epicenter of the disease with more than 90% of cases in mid-April 2020) and the progressive decentralization and capacity building of other national and regional laboratories (from May 2020 onwards) (35). Despite this, a slow pace in testing intake was observed, with the delivery of results taking 72 hours or more instead of the 48 hours recommended (36). As part of the “Stop COVID-19 in 60 Days” initiative (May 2020), mobile sampling units were set up in Conakry, with the involvement of community health workers (37). This was aimed at alleviating community reluctance and breaking the transmission chain at an accelerated pace (37). Finally, screening units were set up at bus stations for mandatory testing (free of charge) of all passengers leaving Conakry (38). A similar scheme was also implemented for foreign travelers in late July 2020, but with a payment of USD 65 per test.

Second, the strategy for case isolation originally included two former Ebola treatment centers (one in Conakry and the other in the countryside). These centers were made operational in early February 2020 [Ministère de la Santé: (21)]. However, their combined capacity did not exceed 73 beds. In addition, a video of a press conference published on the ANSS website (March 16, 2020), revealed that the quality of the facilities was considered sub-optimal by the first patients, mostly the Guinean elite. This led to rehabilitating the Donka national hospital premises and the transfer of COVID-19 patients there. Nevertheless, the capacity of the Donka hospital was rapidly exceeded by mid-April 2020 with many patients waiting in the hospital's corridors to receive their first care (15). Consequently, four other treatment centers were established in Conakry to decentralize COVID-19 care delivery. For instance, in April 2020, following the notification of a wave of contamination in the prisons of Conakry, a treatment center was created at the Alpha Yaya Diallo military camp to primarily receive prisoners of civil rights. Additionally, intensive care services of the Donka and other private hospitals were requested for managing complicated COVID-19 cases.

In late April 2020, decentralization of care at the household level was adopted but only officialised in late May 2020 through the “Stop COVID-19 in 60 Days” initiative. This implied that COVID-19 patients would be treated at home, without the need to be admitted to a treatment center. Unfortunately, this decision turned out to be an opportunity for some actors, especially the elite and senior civil servants, to negotiate their home care with private medical staff, or to resist admission to a treatment center—because of their perceived poor quality. Subsequent deaths of high executive officials—including a minister, a chairman of a public institution and several senior public servants—were reported between May and June 2020 and were seemingly related to their late admission to treatment centers (15). In response to this, an act was introduced by the MoH on 27 July 2020 to prohibit home confinement. This act highlighted that “any person who tests positive would immediately be transferred, either voluntarily or by force, to a treatment center.”

The re-adaptation of isolation strategies was not limited only to the capital, but also the countryside. For instance, in the countryside, care for COVID-19 patients was initially provided at the regional level, meaning that patients diagnosed in health districts were admitted to regional treatment centers. As the virus spread extensively from mid-April 2020 onwards, epidemic treatment centers at district levels, previously used for routine health care provision, were seized and rehabilitated for COVID-19 patients' care.

Third, during the response to this first wave and beyond, the rise of new international players was noted. For instance, the role The Alliance for International Medical Action (ALIMA) was crucial in managing COVID-19 patients. In addition, several initiatives were undertaken by mining companies from China, Russia and the United Arab Emirates (UAE), based in Guinea under bilateral cooperation, during this first wave and beyond. For example, the contribution of the UAE—through Emirates Global Aluminium's Guinea Alumina Corporation—was crucial in building a treatment center in Conakry. It also appeared that three treatment centers located in mining zones in the countryside (Fria and Kindia) were financially and technically supported by Rusal, the Russian aluminum company producer (39). Another illustration was the involvement of a Cuban medical team, including 21 epidemiologists, in the COVID-19 response upon the government's request (40). Table 3 provides a list of actors and their responsibilities, as described in the pandemic response framework.

**Table 3:** Health system actors and their roles in the COVID-19 response.

**Actors**	**Area of responsibilities**	**Roles in the COVID-19 pandemic response**
**Ministry of Health:** ANSS, National Directorate for Major Endemics and Disease Control, National Directorate of Community Health and Traditional Medicine **Technical and Financial partners:** WHO, CDC, IOM	Epidemiological surveillance	• Define surveillance guidelines and technical documents • Define strategies for identifying and monitoring cases and managing surveillance data • Update the definition of cases according to the evolution of the pandemic • Update the areas of intervention of the prefectures at risk; follow up on the collection of cases and contacts
**Ministry of Health:** ANSS, central pharmacy of Guinea, material and equipment service, National Directorate of Pharmacy and Medicines, health department of Conakry, Expanded Programme on Immunization, National Blood Transfusion Center **Ministry for cooperation** **Ministry of defense:** Military Health Service, Customs directorate general **Technical and Financial partners:** World Food Program, WHO, CRS, Red Cross, UNICEF	Logistics	• Define the strategy for the supply • Management of supplies • Coordinate the acquisition of supplies as part of the response against COVID-19 • Carry out an inventory of stock requirements • Define the training programme for logistics personnel
**Ministry of Health**: National Directorate of Laboratories, National Institute of Public Health, National Laboratory of Public Health, National Haemorrhagic Fever Laboratory, CERFIG, UAGCP, CREMS, LABOGUI **Ministry of Education and Scientific Research**: University of Conakry **Technical and Financial partners:** WHO, Pasteur Institute	Laboratory	• Determine the nature of the operating protocols to be used • Validate the laboratories to be integrated into the diagnostic system on the basis of conformity examinations • Determine the way in which sampling is organized • Determine the sample distribution circuit and sites • Organize the validity of the proposed tests • Assess the biosafety and biosecurity conditions of the laboratories • Organizing the management of samples • Organizing the training of laboratory staff at national level
**Ministry of Health:** ANSS, national health promotion service, **Ministry of Communication:** national public and private media **Youth Ministry:** CENAFOB **Ministry of social welfare** **Prime minister's cabinet:** religious affairs secretariat **Technical and Financial partners**: UNICEF, USAID, UNFPA	Communication and social mobilization	• Define communication strategy • Design communication plan with a budget • Organize communication and social mobilization activities • Coordinate interventions in the media and communities • Involve leaders and other personalities in favor of the fight • Mobilize and involve community platforms and provide advice to the strategic committee • Collect and manage rumors
**Ministry of Health:** Nationals hospitals of Donka, Ignace Deen and Sino-Guinean friendship, National Directorate of Health Facilities **Ministry of Defense:** Civil defense **Technical and Financial Partners:** MSF, ALIMA, JHPIEGO, OMS, France Expertise, Red Cross, International Federation of the Red Cross, IOM	Clinical care	• Identify and assess management sites according to the evolution of the epidemic • Produce and update management protocols according to the evolution of knowledge about the disease • Assess therapeutic needs • Define the Infection control and prevention protocol in the context of the response to COVID-19 • List the consumable drugs and equipment that can be used in the response to COVID-19 • Establish guidelines for transporting and burying bodies • Produce the training plan for management personnel in the response to COVID-19
**Ministry of Health:** ANSS, Head of the cabinet, bureau of strategy and development, financial affairs division, human resources department public procurement **Ministry of Budget** **Ministry of Investment** **Technical and Financial Partners:** World Bank, European Union	Finances	• Carry out the financial assessment of the needs for the implementation of the national response plan • Prepare the files and requests for the financing of the activities; to draw up the financial table of the response • Draw up the manual of procedure for the management of resources according to the conventions and national regulations • Give the status of the mobilization and execution of the national plan budget
**Inter-departmental committee** including the Prime Minister's cabinet **Strategic committee:** MoH, ANSS, Technical and Financial Partner (WHO, CDC, IOM, UNICEF, etc.) **Scientific committee:** 17 members including Academics and Researchers	Coordination	• Provide a framework for consultation, guidance and decision-making for the response to the pandemic • Coordinate activities related to the COVID-19 response

Finally, the emergence of several local initiatives was noted during this phase, including local production of hydroalcoholic solutions by several training and research institutions, local manufacture of spraying devices and their installation at the airport and COVID-19 treatment centers, and manufacture of devices for automatic recording of COVID-19 patients' parameters (temperature, blood pressure, heart rate, etc.). Another initiative, not the least, was the self-declaration of elites suffering from COVID-19 on social media, and their admission to public hospitals. This was of particular importance as it occurred in a period when the elites were criticized for not accepting admission to treatment centers. Furthermore, several sewing workshops engaged in producing traditional masks (made from cloth) and sold them at the government-set price (USD 0.50 per unit). Lastly, therapeutic trials based on phytomedicines were undertaken in COVID-19 treatment centers (41).

##### Mitigation measures overlooking some local realities

On 2 April 2020, Guinea adopted an economic contingency plan to mitigate the potential effects of the pandemic. This plan has three main components: (1) the health component, (2) the social component and (3) the economic component (Office of the Prime Minister: Plan of economic response to the health crisis of COVID-19, April 2020). The health component was aimed at financing the rollout of the pandemic preparedness and response and strengthening the health system capacities (infrastructure, equipment, etc.). The social component included cash transfers to 1.6 million vulnerable people identified nationwide; the full coverage of water, electricity and transport bills for 3 months; and the provision of prevention equipment to almost 200 000 targeted households (Office of the Prime Minister: Plan of economic response to the health crisis of COVID-19, April 2020). In addition to this, many companies, public departments, social and political movements initiated the distribution of facemasks and handwashing devices to the population as their contribution to the pandemic control. The economic component comprised measures that support the private sector including a three-month postponement of fiscal taxes payment for companies and an exemption from duties and taxes on health equipment dedicated to the COVID-19 response (Office of the Prime Minister: Plan of economic response to the health crisis of COVID-19, April 2020). This policy, estimated at USD 400 million, had to be financed through two mechanisms: the national response funds and the contribution of bi- or multilateral partners (Office of the Prime Minister: Plan of economic response to the health crisis of COVID-19, April 2020). The national funds were sourced from the withholding taxes on fuel prices, savings from the deferral of external public debt servicing and funds reallocation from the MoH and other ministries (Office of the Prime Minister: Plan of economic response to the health crisis of COVID-19, April 2020).

In April 2020, health system actors noticed a sharp decrease in health care utilization between January and March 2020. This was reportedly linked to the stock out of medical supplies, including protective equipment and fear among health personnel and population to provide or seek care (42). To mitigate this, a “health system resilience strategy for the continuity of care” was developed in May 2020 (42). This strategy was layered in five specific objectives including ensuring infection prevention and control among healthcare providers and beneficiaries, availability of health services, and effective communication for patients and their caregivers. The cost of rolling out this strategy was estimated at USD 11 million.

In the education sector, online courses on the national public media were organized between April and July 2020, for candidates for national exams. This was initiated to mitigate the effects of the closure of schools during the first wave. Finally, in many public services, telework and the lay-off of contractual workers and trainees were undertaken to limit the virus from spreading.

However, several questions were raised on the equity of this teaching method. Many media questioned, for instance, how students from poor families managed to follow online courses. Some other media also questioned how the social component of the economic contingency plan was rolled out in the context of political turmoil.

### Phase 3: Alleviation of restrictive measures: Politics flexibility to social demands, 15 July 2020 to 31 January 2021

On 15 July 2020, President Alpha Conde, in an address to the nation, announced the alleviation of certain restrictions: the gradual reopening of air and land borders in accordance with the reciprocity measures between countries; the curfew was also pushed back from midnight to 4 a.m. (as against 9 p.m. to 5 a.m. initially). On 17 July 2020, a protocol for the reopening of air borders was adopted by the Ministries of Transport and Health. This protocol describes the arrangements in place for health control at air entry points (e.g., certificate of RT-PCR test negativity valid for 5 days) and community-based follow-up of travelers' contacts (e.g., self-quarantine for 14 days) in Guinea.

This alleviation of restrictive measures came on the eve of a series of statements by some religious leaders, asking the authorities to facilitate the celebration of “The Tabaski” scheduled for 31 July 2020. The decree of isolating the capital was also repealed to facilitate the movement of people to the countryside. During this period, the country was still confronted with the first wave of the pandemic with increases in confirmed and hospital fatalities cases, at least until September 2020 (June 2020: 1645 cases, 8 deaths; July 2020: 1895 cases, 15 deaths; August 2020: 2009 cases, 13 deaths; September 2020: 1297 cases, 7 deaths). Despite this, the government pursued the adoption of pandemic alleviation measures including gradual schools and public places reopening (October 2020), the launch of the presidential election campaigns (September to October 2020) and the holding of presidential elections (18 October 2020).

The ANSS, in response to the intensification of mass movement in the country, adopted “active case finding” as a strategy for interrupting the COVID-19 transmission chain (October 2020). A key element of this strategy consisted of a campaign for systematic screening of 80% of high-risk groups including patients with influenza-like syndromes, febrile patients with a negative RDT for malaria, health workers, security forces and people over 60 years old.

In September 2020, a bi-annual immunization plan was developed and adopted in December 2020, with the objective of vaccinating 95% of targeted people (18 years and older) before January 2022. In December 2020, using 60 doses of Sputnik V, Guinea was among the countries that initiated vaccination against COVID-19 early (43). Despite these, the delivery of additional COVID-19 vaccines was not effective until the country had experienced a new wave of the COVID-19 pandemic in February 2021.

### Phase 4: The multiple epidemics period, 1 February to 30 June 2021

By February 2021, the country experienced a new wave of the pandemic; 1,006 confirmed cases and 5 hospital deaths in February 2021, 4,198 cases and 30 deaths in March 2021, and 799 cases and 14 deaths in June 2021. This period coincided with the emergence of Ebola (on 12 February 2021), Lassa Fever (on 18 May 2021), and many other recurrent infectious diseases including measles, yellow fever, poliomyelitis and meningitis (7).

The concomitant emergence of these epidemics overstretched the ANSS, the principal national public health institute, and had an impact on COVID-19 surveillance and response (18, 44). It was noted also that the surveillance and response to recurrent epidemics (measles, meningitis, poliomyelitis, etc.) were neglected in favor of Ebola (7).

Although the national vaccination plan has been in place since December 2020, it was not until 3 March 2021 that the country received the first bulk quantity of vaccines (~152 000 doses of Sinopharm). Consequently, the mass vaccination campaign against COVID-19 was only launched on 5 March 2021. Thereafter, 20 000 doses of Sputnik (8 March 2021), 69 000 doses of AstraZeneca (29 March 2021), 23 000 doses of Sinovac (19 April 2021), 194 000 doses of AstraZeneca (11 April 2021) and 300 000 doses of Sinovac (18 April 2021) were delivered to ANSS. These vaccines were sourced through three main channels: (1) bilateral cooperation—Sinopharm and Sinovac vaccines were purchased by the government from China while Sputnik vaccines from Russia; (2) regional cooperation-−69 000 doses of the AstraZeneca vaccine were offered by the African Union; and (3) the Covax initiative provided 194 000 doses of the AstraZeneca vaccine (7). Despite efforts deployed, the vaccination coverage remained low as of 31 May 2021. Indeed, by this date, only 4% of the target population had received their first dose of the vaccine.

Additionally, reinforcing the response to Ebola and Lassa fever that involved graduates of the CDC-led Field Epidemiology Training Programme (45, 46). Furthermore, some experiences of the COVID-19 response were applied for Ebola control. For example, all national and international workforce deployed in N'zérékoré for the Ebola response were administered an Ebola vaccine.

### Phase 5: The COVID-19 variants period, 1 July to 31 October 2021

**Figure 3:**
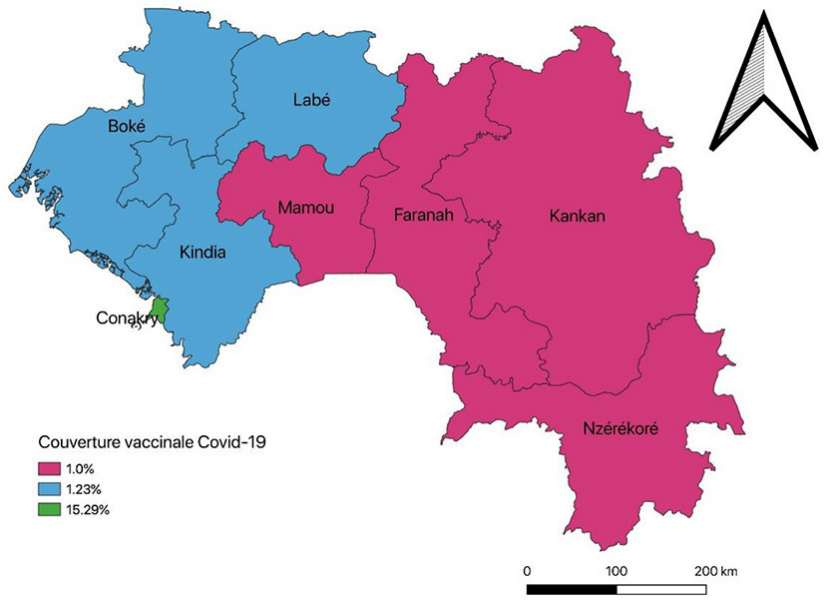
Full vaccination coverage against COVID-19 per administrative regions in the country, August 2021, Guinea.

The news of the end of Ebola (19 June 2021) and the second wave of COVID-19 (29 June 2021) lasted only a few weeks since in July 2021 a new wave started. This wave—the deadliest in the country since the emergence of COVID-19—coincided with the notification of Delta, Alpha, Beta, Gamma and Eta COVID-19 variants in the country (47). This wave bore, respectively, 25 and 59% of COVID-confirmed cases and deaths cases registered in the country from March 2020 to November 2021. The emergence of SARS-CoV-2 variants coincided with low vaccination rate of the population. For example, as of August 2021, only 41% of the 2 900 000 doses of vaccines supplied to Guinea had been used, and full immunization coverage of the population was only 3.56%. This full immunization coverage was five times lower than the 20% projection of the national COVID-19 immunization plan. In addition, strong disparities were noted between the country's health regions in terms of complete vaccination coverage against COVID-19. The complete vaccination coverage of the Conakry health region was 15.29% compared to 1.23% for the Labé, Kindia and Boké regions, and 1% for the Mamou, Kankan, Faranah and N'zérékoré regions (Figure 3).

In response to this, in September 2021, the Guinean government adopted the “accelerated COVID-19 vaccination plan” with the aim of “improving equity of access to vaccines and contributing to the reduction of mortality and morbidity related to COVID-19, through the complete vaccination of 20% of the population before the end of December 2021” (48). This plan was coupled with the adoption of the “vaccination pass” which made movements between cities and access to public services conditional to the presentation of a vaccination card against COVID-19 (47).

This plan was based on 11 acceleration measures, including (1) the use of mobile and semi-mobile strategies (identification of neighborhoods by communes/health districts for sequential immunization), (2) the involvement of certain public and private health centers in immunization and (3) the involvement of civil society associations and corporations in immunization (48). In addition, through this strategy, the supply of COVID-19 vaccines increased from 2.9 to 5.7 million doses between August and November 2021. Also, during the same period, full immunization coverage of the general population increased from 3.56 to 6.95%.

However, the objectives of complete vaccination of 20% of the general population by the end of December were far from being achieved. Similarly, disparities persisted in terms of access to COVID-19 vaccines—on 22 November 2021, full vaccination coverage in Conakry was 25.34%, compared with 3.47% for the Labé, Kindia and Boké regions, and 2.64% for the Mamou, Kankan, Faranah and N'zérékoré regions.

COVID-19-related activities, including vaccination, experienced a slower pace after the military coup of September 2021 as a result of, among others: the freezing of ANSS bank accounts; the reshuffling of management positions at the ANSS and the MoH; the abolition of the vaccine pass and the lifting of the curfew, and the state of health emergency (49). All these raised questioned on how health system actors will reorganize to pursue the COVID-19 control in Guinea.

## Discussion

This study analyses Guinea's preparedness and response to the COVID-19 pandemic during the first 22 months. This study provides five key learning points for future epidemics and pandemic preparedness and response in Guinea, and beyond. These include, in the pre-epidemic period, the necessity of setting up an epidemic governance framework that is articulated with the country's health system and epidemiological contexts; and the importance of mobilizing emergency funds to support a rapid health system response whenever epidemics or pandemics hit. This study also teaches that each epidemic is unique, and previous exposure to similar ones does not necessarily guarantee population and health system resilience. Moreover, the study analyses that epidemics generate social distress because of the restrictive measures they require for their control, but the excessive securitization of epidemics response is counterproductive. Finally, from a political point of view, decision-making for epidemic control is not always disinterested; it is sometimes rooted in political computations, and health system actors should learn to cope with it while at the same time, safeguard trusted and efficient health system responses.

First, the analysis of pandemic preparedness reveals an early response, almost 47 days before the virus was introduced into Guinea. This early response was coordinated by the ANSS, the former Ebola coordination body, which has strengthened its leadership in the response to recurrent epidemics including anthrax, measles and yellow fever from 2017 onwards. This recent role of ANSS in the response to epidemics in Guinea had implicitly played its value, particularly in obtaining support from several actors, including decision-makers and development partners, for the coordination of preparedness and response to COVID-19. Despite this, however, we noted tensions between several health system actors when it came to formally assigning, through a presidential decree, the coordination of the response to the ANSS. These tensions were likely entertained by the absence, before COVID-19, of a formal framework for epidemic governance, with a clear distinction between the roles and responsibilities of the actors. The difficulties in coordinating the COVID-19 response mechanisms at the beginning of the pandemic might explain the increase in incidence cases of disease during this period in Guinea, compared with west African countries like Liberia, Sierra Leone, Mali, and Burkina Faso (8, 12). This is why we argue that national stakeholders should formalize epidemic governance framework, in pre-epidemic period, in order to ensure effective coordination of epidemic or pandemic response. Such a governance framework should take into account local specificities.

In Guinea, for example, two options can be envisaged with their pros and cons. A first option would be to establish the ANSS as the leading organization for the coordination and implementation of epidemics and pandemics preparedness and response interventions. Such a measure, because of its centralized nature, would facilitate decision making and operationalisation of health interventions in the face of epidemics or pandemics. However, as noted during this study, the concurrent emergence of epidemics would substantially reduce the response capacity of such an arrangement (7, 44). Also, such a vertical response mechanism would raise questions about the sustainable strengthening of the health system, including routine immunization services and interventions since the latter has not yet been integrated with COVID-19 immunization in Guinea. Also, in a context of political instability—with its implication for change in leadership positions in national institutions—the risk that institutional memory for epidemics preparedness and response is altered could be high. Specially, because with the recurrence of epidemics and pandemics in Africa, the mobility of such experienced health workers in managing epidemics could also be high.

A second option in Guinea would be to decentralize epidemics and pandemics response functions. For example, in the pre-epidemic period, the DNELM could be responsible for routine surveillance of diseases with epidemic potential. In the event of an epidemic or pandemic, this surveillance role could be devolved to the ANSS while the INSP would be responsible for their diagnosis. The EPI/DNELM would be responsible for the vaccination pillar in the response to epidemics. Such a mechanism therefore implies setting up the ANSS as an “agence de veille sanitaire” in the pre-epidemic period. However, such a scheme would give the advantage to several national institutions to test their capacities during epidemics and draw lessons for their continuous strengthening.

Second, this study highlights the unpredictability of epidemics including their occurrence in a difficult socio-political and economical context. In Guinea, the pandemic occurred in Guinea in a period when donor countries were confronted with an acute phase of the disease. Importantly, the COVID-19 pandemic also occurred in the middle of an electoral process for the renewal of legislative and presidential institutions. In this context, despite the anticipation of the response by health system actors, the implementation of the pandemic preparedness and response interventions was difficult, due to among others the low mobilization of financial resources to support the COVID-19 response strategies. This finding raises the question on the need of putting in place sustainable funding mechanisms for improving national health system readiness toward epidemic-prone diseases. One way of doing this may be creating, in pre-epidemic situations, an emergency fund that can be used by health system actors as soon as an epidemic-prone disease occurs on the national territory. Such funding mechanisms could avoid unnecessary delays in responding to emergencies.

Third, this study also analyses that each epidemic is unique, and previous exposure to similar ones does not necessarily guarantee population and health system resilience. In 2014, Guinea was the epicenter of the largest and deadliest Ebola outbreak in human history. This outbreak triggered several health system reforms to improve the national health system resilience. These efforts yielded positive results in the rapid response to the recurrent epidemics in Guinea, including the 2016 Ebola outbreak (7). However, our findings showed that the country was not well-prepared to deal with the COVID-19 pandemic. First, Ebola treatment centers were proven inappropriate to manage COVID-19 cases due to lack of adequate equipment—for example, lack of oxygen tanks—for COVID-19 treatment. Second, the testing capacity of national reference laboratories were very limited, given the high infectivity of COVID-19 compared to Ebola. Similarly, the bed capacities of Ebola treatment centers were very limited during the early phases the COVID-19 pandemic. Third, the health services utilization was compromised during the first wave of the pandemic due to a lack of personal protective equipment for health workers, and people's fear of visiting health facilities. All of these point to the fact that the Ebola experience did not guarantee effective preparedness and response to the COVID-19 pandemic. Delamou et al. in their rapid assessment of health system preparedness and response to the COVID-19 pandemic undertaken in 13 health districts in Guinea, reported that most of healthcare facilities visited did not have intensive care units (95%), oxygenators (98%), and respirators (94.4%) (50).

Fourth, the finding shows that epidemics generate social distress because of the restrictive measures they require for their control, but their excessive securitization is counterproductive. The securization of the COVID-19 pandemic response has been documented to negatively impact populations health with implication on injuries and deaths as results of policy interventions, but also an increase in their resistance to government response measures (7).

Fifth, the analysis of the COVID-19 pandemic response process in Guinea reveals that decision-making was sometimes underpinned by political considerations. At times, COVID-19 was used by politicians as a springboard to push through decisions that were favorable to them. For example, bans on mass gatherings or movements on the eve and aftermath of elections were surely taking as a dissuading measure for political protests. Such situations have also been reported in other countries such as France (51). However, health system actors should learn to cope with it while at the same time, safeguard trusted and efficient health system responses. In Guinea, health system actors adapted to this situation by emphasizing the importance of facemask wearing and compliance with barrier measures during political campaigns, appealing to political leaders for their responsibility to enforce health guidelines related to COVID-19, but also by undertaking active case finding and systemic testing among high-risk groups following periods of intensified mass movements.

Furthermore, referring to Kruk definition of resilient health system, this study analyzed that the Guinean health system resilience capacities have been improved (52). First, the Guinean health system actors adopted an earlier response, about 47 days, in face of the SARS-CoV-2 introduction on the national territory, notably through the planning of epidemiological surveillance, and case management. This result could be explained by the experience heath system actors have acquired in managing the 2014–16 Ebola epidemic, the gains of the post-Ebola health system reforms that led to the establishment of a central public health emergency operation department networked with 38 health districts and the training of biosecurity workforce (45). Authors have reported that exposure to major epidemics in the past is associated with earlier responses, especially in implementing epidemiological surveillance strategies (53). In Liberia and Sierra Leone, the two other West-African countries severely affected by Ebola in 2014–16, rapid readiness of health systems have also been reported (54, 55). Second, the declaration of the COVID-19 pandemic triggered the undertaking and implementation of several innovative approaches in Guinea. These initiatives include a phytomedicine therapeutic trial; manufacturing of hydroalcoholic solutions and traditional facemasks; and COVID-19-patient monitoring and spraying machines. These initiatives have certainly helped the country to overcome the global challenges of health resources (e.g., masks, hydroalcoholic solutions), especially observed in developing countries, for the control of the acute phase of the pandemic (56, 57). Some authors define attitudes that help societies to resist shocks as an indication of improved collective resilience (58, 59). Third, the COVID-19 pandemic response in Guinea acknowledge the rise of new players in the health system, compared with Ebola in 2014/2016. Despite the positives discussed, some challenges have been noted in the COVID-19 pandemic response processes in Guinea. This include the indirect effects of the COVID-19 pandemic control measures on populations socio-economic and psychological wellbeing. For instance, increases in transportation fare and loss of income for many private institutions in Guinea such as restaurants, bars and hotels were noted as results of, unnecessarily prolonged restrictive measures. Authors in South Africa have reported that the prolonged effects of restrictive measures such as lockdowns have resulted in acute panic, depression, anxiety and social unrest (60).

The present review has some limitations. First, the methodological design of this study—scoping review—did not allow any quality (or risk of bias) assessment of included studies. This could limit the internal validity of the findings reported in the present study. Second, this review covers the period between January 2020 and November 2021. Given the highly dynamic and changing prospects of the COVID-19 pandemic, and the high political instability in Guinea, conclusion of the study may not be applicable to next phases of COVID-19 response. However, this study utilizes diverse data sources to allow a better understanding of the responses from the government and other civil actors in the country, during the 22 first months of the pandemic. It therefore provides insights on challenges health system actors have been confronted with, and proposed solutions for an improved preparedness and response strategies of future epidemics and pandemics in Guinea.

## Conclusion

This scoping review aimed to assess the response triggered in Guinea between January 2020 and November 2021. It provides several learning points for future epidemics and pandemics preparedness and response in Guinea, and beyond. These learning points include the necessity of setting up, in the pre-epidemic period, an epidemic governance framework that is articulated with the country's health system and epidemiological contexts; the importance of mobilizing, during pre-epidemic period, emergency funds for a rapid health system response whenever epidemics hit; each epidemic is a new experience as previous exposure to similar ones does not necessarily guarantee population and health system resilience; epidemics generate social distress because of the restrictive measures they require for their control, but their excessive securitization is counterproductive; and that decision-making for epidemic control is not always disinterested, from a political perspective, and that health system actors should learn to cope with it while safeguarding trusted and efficient health system responses. Health system actors anticipated the response to the COVID-19 pandemic and (re-) adapted response strategies as the pandemic evolved. There is a need to work toward rethinking the epidemics governance framework and funding mechanisms in Guinea for improved health system efficacy toward their management.

## Author contributions

The study protocol was developed by DK, FK, and AD and reviewed by WV and IA. DK and FK did the data analysis, performed the literature search, screening, and selection of articles. The first draft of the manuscript was written by DK. The manuscript was critically reviewed by WV, J-PD, and IA. All authors were involved with interpretation, read, and agreed to the final version of this manuscript.

## Funding

This study was funded by the International Development Research Centre (IDRC), Canada through the Grant number (CATALYSE PROJECT 109479-001). The funder had no role in study design, data collection and analysis, decision to publish, or preparation of the manuscript.

## Conflict of interest

The authors declare that the research was conducted in the absence of any commercial or financial relationships that could be construed as a potential conflict of interest.

## Publisher's note

All claims expressed in this article are solely those of the authors and do not necessarily represent those of their affiliated organizations, or those of the publisher, the editors and the reviewers. Any product that may be evaluated in this article, or claim that may be made by its manufacturer, is not guaranteed or endorsed by the publisher.
